# Systematic Review of Post-Viral Delayed Inflammation Associated with Hyaluronic Acid Dermal Fillers

**DOI:** 10.3390/medicina61101764

**Published:** 2025-09-29

**Authors:** Lorena Bhatia, Saja Al Rekabi, Audra Janovskienė, Inesa Stonkutė, Dainius Razukevičius, Justina Stučinskaitė-Maračinskienė

**Affiliations:** 1 Faculty of Odontology, Medical Academy, Lithuanian University of Health Sciences, J. Lukšos-Daumanto 2, LT-50106 Kaunas, Lithuania; sajaalrekabi@gmail.com (S.A.R.);; 2 Department of Maxillofacial Surgery, Medical Academy, Hospital of Lithuanian University of Health Sciences, Eiveniu 2, LT-50161 Kaunas, Lithuania; audrjano0210@kmu.lt (A.J.); dainius.razukevicius@lsmu.lt (D.R.);

**Keywords:** hyaluronic acid, dermal fillers, delayed inflammatory reactions, viral infection, COVID-19 vaccination, vaccination, SARS-CoV-2 vaccine, esthetic medicine, immunological response

## Abstract

*Background*: Hyaluronic acid (HA) dermal fillers are among the most widely used injectable materials in esthetic medicine. They are generally safe, but delayed inflammatory reactions (DIRs) have been observed, particularly after viral infections or vaccinations. Such events have raised questions about the role of immune activation in filler-related complications. *Objective*: This review examined the available literature on DIRs to HA fillers that occurred in the context of viral illness or immunization, with attention to how these reactions present and how they are managed. *Methods*: A systematic search was carried out in PubMed, ScienceDirect, ClinicalKey, and Google Scholar between October and November 2024. Only human case reports and case series were included. The protocol was registered in PROSPERO (CRD420251030918), and study quality was assessed using the Newcastle–Ottawa Scale. *Results*: Six publications met inclusion criteria: four case series and two case reports, describing 25 women between 22 and 65 years of age. Patients developed swelling, erythema, angioedema, or, in severe cases, marked facial edema after HA filler injections, with symptom onset ranging from several hours to several weeks following viral exposure. Corticosteroids and hyaluronidase were the most common treatments, though milder cases sometimes resolved without intervention. Study quality varied, with some reports providing limited detail on patient characteristics and follow-up. *Conclusions*: DIRs associated with viral infections or vaccinations remain uncommon but clinically relevant complications of HA filler use. Limited case-based evidence indicates potential effectiveness of corticosteroids and hyaluronidase, though management practices remain inconsistent. Larger prospective studies are needed to clarify underlying mechanisms and to establish standardized guidelines for treatment.

## 1. Introduction

Over the past decade, injectable hyaluronic acid (HA)-based dermal fillers have emerged as a foundational modality within the domain of minimally invasive esthetic medicine. These agents are extensively employed for the correction of age-related soft tissue deficits, enhancement of facial contours, and restoration of dermal volume loss [1]. The rising clinical adoption of HA fillers is largely attributed to their favorable risk-benefit profile, biocompatibility, and their ability to address facial structural alterations associated with senescence, trauma, and systemic pathologies.

According to recent global market analyses, the valuation of the HA dermal filler sector reached an estimated USD 4.08 billion in 2023, with projections indicating a sustained expansion at a compound annual growth rate (CAGR) of approximately 10.5% from 2024 through 2030. This surge is driven by increased public demand for non-surgical cosmetic interventions across diverse demographic segments, including the male population. Nonetheless, stringent pharmacovigilance frameworks and regulatory barriers in Western markets, particularly the United States and European Union, continue to moderate the pace of product innovation and clinical deployment [2,3].

The clinical efficacy of HA fillers is underpinned by their viscoelastic properties, hydrophilicity, and capacity for reversible tissue augmentation. Mechanistically, HA serves as a transient volumizing agent that mitigates rhytides, restores facial symmetry, and ameliorates the visible manifestations of cutaneous aging. These esthetic sequelae are primarily the result of multifactorial processes including craniofacial skeletal remodeling, gravitational ptosis, redistribution of facial adipose compartments, and progressive dermal matrix degradation [4,5].

Ideal dermal filler biomaterials are characterized by long-term safety, biodegradability, minimal migratory potential, cost-effectiveness, and reversibility in the event of adverse outcomes. Commercially available HA formulations—such as Hylaform^®^, Restylane^®^, Juvéderm Ultra^®^, and Juvéderm Ultra Plus^®^—exhibit variable physicochemical characteristics, notably in terms of polymer concentration, viscoelastic behavior, and degree of crosslinking, which directly influence their clinical performance and longevity [6,7].

From a biochemical standpoint, HA is an anionic, non-sulfated glycosaminoglycan integral to the extracellular matrix, composed of alternating units of D-glucuronic acid and N-acetyl-D-glucosamine linked via β-1,4-glycosidic bonds [8,9]. To enhance in vivo durability, exogenous HA utilized in dermal fillers undergoes crosslinking—a process that establishes covalent bridges between polymer chains, transforming the native viscous solution into a stable, cohesive gel with improved resistance to enzymatic degradation [10,11].

Despite advances in formulation science, increased structural complexity in HA fillers has been correlated with a heightened risk of delayed hypersensitivity reactions [12,13]. These adverse events—characterized by localized induration, erythema, and edema—typically manifest as T-cell-mediated immune responses rather than immediate antibody-driven mechanisms [14,15]. Recent evidence suggests that such reactions may be precipitated or exacerbated by systemic viral infections, including those with influenza-like presentations and SARS-CoV-2 [16,17].

This study aims to systematically analyze the extant scientific literature concerning the immunological sequelae of HA dermal filler use, with particular emphasis on delayed inflammatory responses following exposure to viral pathogens.

## 2. Materials and Methods

### 2.1. The Protocol for the Systematic Review

This systematic review was conducted in accordance with the methodological standards set forth by the Preferred Reporting Items for Systematic Reviews and Meta-Analyses (PRISMA) guidelines. To enhance methodological transparency and reduce the risk of duplication, the review protocol was prospectively registered with the International Prospective Register of Systematic Reviews (PROSPERO; registration number CRD420251030918). The research question was developed using the PICO framework, which specifies the Population, Intervention, Comparator, and Outcome elements to ensure a structured and clinically meaningful line of inquiry. A comprehensive summary of the PICO components is provided in Table 1.

### 2.2. Types of Publications

This review encompasses clinical case reports and case series involving human subjects who underwent facial administration of hyaluronic acid-based dermal fillers and subsequently exhibited delayed-onset inflammatory responses temporally associated with viral infections.

### 2.3. Types of Studies

The final selection comprised four clinical case series and two individual case reports, each documenting delayed inflammatory reactions following hyaluronic acid dermal filler injections in the context of viral infection.

### 2.4. Information Sources

A comprehensive electronic literature search was conducted across PubMed, ScienceDirect, and ClinicalKey databases between 19 October 2024, and 21 November 2024.

### 2.5. Article Search Strategy

A targeted electronic literature search was conducted across PubMed, ScienceDirect, ClinicalKey, and Google Scholar, adhering to PRISMA guidelines, to identify all potentially eligible studies. The specific keyword combinations employed during the search process are detailed in Table 2. Following reviewer feedback, the search was expanded to explicitly incorporate terms related to “vaccination” and “COVID-19 vaccine” to ensure comprehensive coverage of the literature.

### 2.6. Inclusion and Exclusion Criteria

Scientific studies were selected for the systematic review according to the inclusion and exclusion criteria listed in Table 3. As predefined in our PROSPERO registration, only case reports and case series providing original clinical data were eligible. Higher-level syntheses such as scoping reviews were excluded to avoid redundancy with already included primary sources.

### 2.7. Selection of Studies

Upon completion of the database search, all identified citations underwent systematic screening in accordance with predefined inclusion and exclusion criteria. The initial phase involved evaluating study titles and abstracts to rapidly eliminate non-eligible records. For citations that appeared potentially relevant, full-text manuscripts were retrieved for comprehensive assessment. This evaluation considered factors such as study design, patient population, intervention characteristics, and outcome measures. Through this tiered appraisal process, only studies meeting all methodological and clinical relevance thresholds were incorporated into the final systematic review dataset.

### 2.8. Population

This analysis includes studies involving female patients aged 22 to 65 years who received hyaluronic acid dermal filler injections in the facial region. The primary focus of the included studies was on cases in which patients developed delayed inflammatory reactions subsequent to a viral infection or COVID-19 vaccination following such treatments.

### 2.9. Sequential Search Strategy

All retrieved articles were independently evaluated to determine compliance with the predefined inclusion and exclusion criteria. Following the initial search, abstracts were screened for relevance to the research question, and studies deemed unsuitable at this stage were excluded. Full-text versions of the remaining eligible articles were then obtained and subjected to a comprehensive review to confirm adherence to the established methodological and thematic parameters.

### 2.10. Risk-of-Bias Assessment

The risk of bias for the included case reports and case series [12,13,18,19,20,21] was assessed using the Newcastle–Ottawa Scale (NOS), a validated tool for evaluating the methodological quality of non-randomized studies. The NOS examines three primary domains: case selection, outcome assessment, and adequacy of follow-up, with potential sources of bias considered within each category. Studies are rated using a star (*) allocation system, in which a higher number of stars reflects superior methodological quality.

## 3. Results

### 3.1. Study Selection

**Figure 1:**
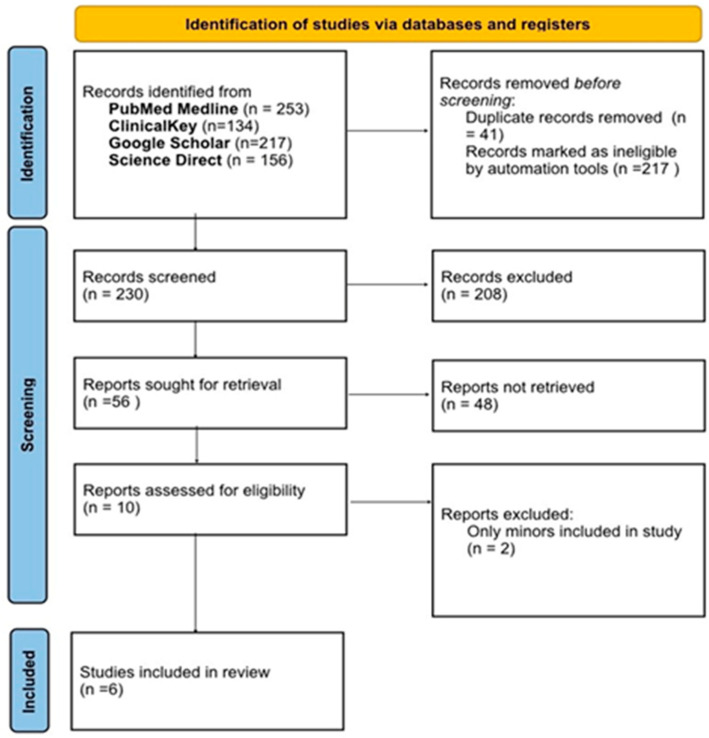
PRISMA flow diagram.

The database search, conducted in PubMed Medline, ClinicalKey, Google Scholar, and ScienceDirect using predefined keywords, initially identified 760 records. Many were excluded at the title and abstract level due to irrelevance, insufficient data, or duplication. After this preliminary screening, 56 studies remained. Of these, 10 met the criteria for full-text review. Ultimately, six studies satisfied all predefined inclusion and exclusion requirements (Table 2) and were incorporated into the review. These publications were released between 2019 and 2022. The search process and study selection are summarized in the PRISMA flow diagram (Figure 1).

### 3.2. Quality Assessment of the Included Studies

Risk of bias for the four case series and two case reports [12,13,18,19,20,21] was assessed using the Newcastle–Ottawa Scale (NOS), which evaluates non-randomized studies across three domains: case selection, outcome assessment (including bias), and follow-up adequacy. Scores of 7–9, 4–6, and 0–3 were interpreted as high, moderate, and high risk of bias, respectively. Munavalli GG et al. [12] and Calvisi L. et al. [19] scored 7 (high quality); Michon A. et al. [18] and Savva D. et al. [20] scored 5, and Beamish IV et al. [21] scored 4 (all moderate quality); Turkmani MG et al. [13] scored 3 (high risk of bias) (Table 4).

### 3.3. Characteristics of Included Studies

Six studies were included in this systematic review [12,13,18,19,20,21], comprising four case series and two case reports. Across these publications, a total of 25 female patients were analyzed, aged 22 to 65 years, with a mean age of 41.3 years. Participant and study characteristics are summarized in Table 5.

All patients described in the included studies were women between 22 and 65 years of age who had undergone hyaluronic acid dermal filler injections. Some reports focused on a single case, while others presented a series of patients. In each study, the individuals experienced delayed inflammatory reactions after filler administration. These reactions were either temporally associated with a viral infection or occurred without any identifiable trigger. A detailed breakdown of cases and their characteristics is shown in Table 6.

Across the included studies, a range of delayed inflammatory reactions following hyaluronic acid dermal filler treatment was documented. Munavalli GG et al. (2021) [12] described four patients, most of whom had recently received a COVID-19 vaccination, presenting with facial swelling, edema, and erythema. Symptom onset ranged from 12 h to two weeks, and treatment with corticosteroids and hyaluronidase resulted in improvement [12]. Michon A. et al. (2021) reported two patients who developed swelling and erythema after viral infection; symptoms appeared within one to several days, resolving spontaneously in one case and after hyaluronidase administration in the other [18]. Calvisi L. et al. (2022) analyzed three patients, all vaccinated against COVID-19, who developed swelling and angioedema approximately five days later; all achieved complete resolution following prednisolone therapy [19]. Savva D. et al. (2021) documented a single case of lip swelling appearing within one to two days and resolving with methylprednisolone [20]. In a larger series, Turkmani MG et al. (2019) described 14 patients who developed flu-like illness accompanied by swelling, with an average onset of three to five days, treated with prednisolone and hyaluronidase [13]. Finally, Beamish IV et al. (2022) reported one patient with severe swelling of the jaws and lips six weeks post-treatment; intravenous diphenhydramine and dexamethasone achieved complete resolution [21].

## 4. Discussion

This systematic review synthesizes evidence from six studies comprising 25 female patients aged 22–65 years who developed delayed inflammatory reactions (DIRs) following hyaluronic acid (HA) dermal filler treatment in the context of viral infection or vaccination. All patients in the included studies were women. While this likely reflects the demographics of dermal filler use, the possibility of sex-related immunological differences cannot be excluded. For example, women have been shown to mount stronger innate and adaptive immune responses, which could theoretically predispose to heightened inflammatory reactions. This warrants caution in generalizing findings to male populations. Across all reports, a consistent association emerged between immune activation from viral exposure and localized inflammatory responses at filler sites.

The clinical spectrum was broad, ranging from localized edema and erythema to angioedema and severe maxillofacial swelling. Symptom onset varied markedly—from 12 h to six weeks—suggesting multifactorial triggers that may involve individual immunological profiles, filler composition, and pre-existing immune sensitivities. The heterogeneity in latency periods also underscores the likelihood of both immediate and delayed immunopathological pathways. Age was not identified as a significant risk factor.

Treatment strategies were heterogeneous, though corticosteroids and hyaluronidase predominated. Corticosteroid use, particularly prednisolone or methylprednisolone, was the primary intervention in moderate-to-severe cases, while hyaluronidase was administered in cases with persistent swelling or nodularity. Antihistamines (e.g., diphenhydramine) and adjunctive antibiotics (e.g., doxycycline) were reported in select cases. Notably, spontaneous resolution occurred in some mild cases, reinforcing the potential for a graded, severity-based management algorithm.

The findings reinforce immunological models proposing that viral antigens or adjuvants may act as secondary triggers for inflammatory responses at previously treated dermal filler sites. This aligns with prior hypotheses on immune complex deposition, delayed-type hypersensitivity, and filler biofilm activation as plausible mechanisms. However, definitive pathophysiological pathways remain unconfirmed.

From an immunological perspective, vaccines and viral infections act as potent immune stimulants through activation of Toll-like receptors, antigen-presenting cells, and subsequent cytokine cascades, particularly IL-1β, IL-6, and TNF-α. These pathways may enhance recognition of filler material as foreign or reactivate low-grade filler-associated biofilms, leading to localized inflammation. Furthermore, vaccine adjuvants are designed to boost immune responses, which may inadvertently contribute to delayed hypersensitivity at filler sites. Such mechanisms underscore the interplay between systemic immune activation and localized filler-related DIRs.

Several limitations must be acknowledged. The cumulative sample size is small, with substantial variability in data reporting, particularly regarding symptom onset and treatment outcomes, hindering direct comparison across studies. Lack of standardized outcome measures and limited follow-up preclude robust conclusions regarding long-term prognosis. Furthermore, absence of detailed demographic and immunological profiling restricts risk stratification.

It is important to emphasize that all included studies were case reports or case series, which inherently carry a high risk of bias. While the Newcastle–Ottawa Scale was applied for quality assessment, its use in this context is limited, as it was developed primarily for observational studies. No prospective or controlled studies are currently available, and therefore, the conclusions of this review must be regarded as preliminary and hypothesis-generating rather than definitive.

Future research should prioritize multicenter prospective studies with standardized definitions of DIRs, uniform treatment protocols, and extended follow-up. The identification of predictive biomarkers—such as HLA genotypes, baseline inflammatory markers, or specific immune signatures—may facilitate targeted preventive strategies. Given the global prevalence of HA filler use and the likelihood of viral exposure, establishing evidence-based clinical pathways is critical to optimizing patient safety and treatment outcomes.

## 5. Conclusions

Six studies were reviewed to examine delayed inflammatory reactions (DIRs) after viral infections or vaccinations in patients with hyaluronic acid dermal fillers. Most reactions followed immune activation from COVID-19 infection, COVID-19 vaccination, or influenza-like illness. Corticosteroids and hyaluronidase were the most common treatments, though some mild cases resolved without intervention. The variation in both onset and severity points to the need for clear, standardized guidelines for diagnosing and managing DIRs in this setting.

## Figures and Tables

**Table 1:** PICO Characteristics.

Component	Description
Population (P)	Patients who developed DIRs to hyaluronic acid dermal fillers following virus infection
Intervention (I)	Hyaluronic acid dermal filler injection
Comparison (C)	None
Outcome (O)	Adverse reactions
Focus question	Delayed inflammatory reactions to hyaluronic acid dermal fillers following virus infection

**Table 2:** Summary of keyword combinations.

Date	Database	Keywords Used	Number of Articles Found
19 October 2024	Pubmed Medline	Hyaluronic Acid, Dermal Filler, Delayed inflammatory reactions following HA, DIR after Dermal Fillers following virus infection, Vaccination, COVID-19 vaccine, SARS-CoV-2 vaccine	253
19 October 2024	Science Direct	Hyaluronic Acid, Dermal Filler, Delayed inflammatory reactions following HA, DIR after Dermal Fillers following virus infection, Vaccination, COVID-19 vaccine, SARS-CoV-2 vaccine	156
19 October 2024	ClincalKey	Hyaluronic Acid, Dermal Filler, Delayed inflammatory reactions following HA, DIR after Dermal Fillers following virus infection, Vaccination, COVID-19 vaccine, SARS-CoV-2 vaccine	134
19 October 2024	Google Scholar	Hyaluronic Acid, Dermal Filler, Delayed inflammatory reactions following HA, DIR after Dermal Fillers following virus infection, Vaccination, COVID-19 vaccine, SARS-CoV-2 vaccine	217

**Table 3:** Selection criteria.

Inclusion Criteria	Exclusion Criteria
Case reports, case series.	Studies conducted on animals or in vitro models
Full access to the article is available. Studies in English language.	Any other type of study: Prospective or Retrospective clinical trials, cohort studies, retrospective analyses, randomized controlled clinical trials, meta-analysis.
Studies in English language.	Full access to the article is not available.
Studies published less than 10 years ago.	Articles published in languages other than English.

**Table 4:** Risk-of-Bias Assessment using Newcastle–Ottawa Scale (NOS).

Study	Selection of Cases	Outcome	Bias and Follow-Up	Total Score
Munavalli, GG et al. (2021) [12]	***	**	**	7 stars
Michon A. et al. (2021) [18]	**	**	*	5 stars
Calvisi L. et al. (2022) [19]	***	**	**	7 stars
Savva D et al. (2021) [20]	**	**	*	5 stars
Turkmani MG et al. (2019) [13]	*	*	*	3 stars
Beamish IV et al. (2022) [21]	**	*	*	4 stars

Interpretation of Newcastle–Ottawa Scale (NOS): Good quality: 7 to 9 stars. Fair / moderate quality: 4 to 6 stars. Poor quality: 3 or fewer stars.

**Table 5:** Types of studies and characteristics of participants.

Study	Study Type	Sample Size (Patients, (*n*))	Age Mean SD	Age Range	Gender
Munavalli, GG et al. (2021) [12]	Case Series	4	45	36–51	Females
Michon A. et al. (2021) [18]	Case Series	2	50	39–61	Females
Calvisi L. et al. (2022) [19]	Case Series	3	48.33	40–60	Females
Savva D et al. (2021) [20]	Case Report	1	38	38	Female
Turkmani MG et al. (2019) [13]	Case Series	14	43.5	22–65	Females
Beamish IV et al. (2022) [21]	Case Report	1	23	23	Female

**Table 6:** Detailed study data of included studies.

Study	Patients (*n*)	Type of HA Dermal Filler	Virus Infection/COVID-19 Vaccination	Post Symptoms	Time of Onset of Symptoms	Common Treatment Used	Conclusion
Munavalli, GG et al. (2021) [12]	4	Restylane, Voluma, Juvederm Voluma	(+) (−) (+) (+)	Burning, facial swelling, erythema, periobital edema, body ache	2 weeks 48 h 12 h 24 h	Hylenex injection, Prednisone, doxycycline and oral corticosteroid	All cases except for one experienced DIR, treatment included corticosteroids, hyaluronidase leading to improvement.
Michon A. et al. (2021) [18]	2	Juvederm Volite, Juvederm Voluma	(+) (+)	Facial swelling, erythematous swelling at tear through area	1–2 days Few days	Spontaneously resolved Dissolved filler with Hyaluronidase injection	DIRs developed in both cases, hyaluronidase was administered resulting in complete resolution.
Calvisi L. et al. (2022) [19]	3	Juvederm Ultra, Juvederm Volift	(+) (+) (+)	Swelling in upper lip, angioedema, erythema and edema.	5 days	Prednisolone	All patients experienced DIRs, 2 of them resolved spontaneously and 1 used Prednisolone.
Savva D et al. (2021) [20]	1	HA	(+)	Erythematous edema on both lips	1–2 days	Methylprednisonole	Course of Methylprednisonole was used after DIRs, resulting in resolution within 5 days.
Turkmani MG et al. (2019) [13]	14	Juvederm Volbella, Juvederm Voluma	All patients (+)	Influenza-like illness. (Fever, headache, sore throat, fatigue)	Average 3–5 days	Prednisolone, Hyaluronidase	DIRs developed in all cases, corticosteroids and hyaluronidase was administered resulting in complete resolution.
Beamish IV et al. (2022) [21]	1	HA	(+) (+) (+) (+)	Acute painful asymmetric swelling in maxilla, mandible and lips	6 weeks	Intravenous diphenhydramine, and dexamethasone	DIRs developed post COVID-19 vaccine, antihistamine and dexamethasone were used, leading to improvement.

(+) Virus Infection or COVID-19 Vaccination present. (−) Virus Infection or COVID-19 Vaccination not present.

## Data Availability

The data is contained within this article.

## Abbreviations

HA	Hyaluronic Acid
DIR	Delayed Inflammatory Reactions
SD	Standard Deviation
